# Adverse Obstetric Outcomes after Breast Cancer Diagnosis: An Observational Database Study in Germany

**DOI:** 10.3390/cancers16183230

**Published:** 2024-09-22

**Authors:** Anna Sophie Scholz, Alexandra von Au, Raphael Gutsfeld, Tjeerd Maarten Hein Dijkstra, Dominik Dannehl, Kathrin Hassdenteufel, Markus Hahn, Sabine Hawighorst-Knapstein, Ariane Chaudhuri, Armin Bauer, Markus Wallwiener, Sara Yvonne Brucker, Andreas Daniel Hartkopf, Stephanie Wallwiener

**Affiliations:** 1 Department of Gynecology and Obstetrics, Heidelberg University Hospital, 69120 Heidelberg, Germany; 2 Department of Women’s Health, University Hospital Tuebingen, 72076 Tuebingen, Germany; 3 Department of Health Promotion, AOK Baden-Wuerttemberg, 70188 Stuttgart, Germany; 4 Department of Gynecology, University Hospital Halle (Saale), 06120 Halle, Germany; 5 Department of Obstetrics and Fetal Medicine, University Hospital Halle (Saale), 06120 Halle, Germany

**Keywords:** breast cancer, pregnancy, obstetric outcome, SGA, preterm

## Abstract

**Simple Summary:**

This research analyzed complications during the first pregnancy after breast cancer based on health data in Germany between 2010 and 2020. This study compared pregnancies of 74 women with breast cancer and 222 healthy controls and found no increased risks of preterm delivery, gestational diabetes, hypertensive disorders, and cesarean section to women after breast cancer. But the analyses revealed worse outcomes with a higher risk for small babies, if delivery was in less than 2 years after breast cancer diagnosis.

**Abstract:**

Background/Objectives: Breast cancer may negatively affect later pregnancy and childbirth. We aimed to analyze the impact of previous breast cancer on obstetric outcomes in postdiagnosis pregnancies. Methods: Insurance claims data in Southern Germany were used to identify breast cancer (BC) survivors with at least one subsequent delivery after cancer diagnosis between 2010 and 2020. In total, 74 BC survivors were compared to 222 age-matched controls with frequency matching on their age at their postdiagnosis delivery. Results: Endocrine therapy was associated with a significantly lower probability of birth compared to BC survivors without endocrine therapy (HR 0.36; 95% CI 0.18–0.53; *p* < 0.0001). The risks of preterm birth, low birth weight (LBW), gestational diabetes, hypertensive disorders, and cesarean section were not significantly increased among BC survivors compared to healthy controls. BC survivors were at an increased risk for a small-for-gestational-age (SGA) fetus (OR 3.24; 95% CI 1.17–8.97, *p* = 0.03). Delivery in less than 2 years after diagnosis increased the risk for SGA (OR 5.73; 95% CI 1.37–24.02, *p* = 0.03) and LBW (OR 4.57; 95% CI 1.32–15.87, *p* = 0.02). Conclusions: Our findings are encouraging regarding the risks of preterm delivery, gestational diabetes, hypertensive disorders, and cesarean section to women who consider pregnancy after BC. Delivery in less than 2 years after diagnosis was associated with an increased risk for SGA and LBW.

## 1. Introduction

Worldwide, breast cancer represents the most commonly diagnosed cancer type among women and is the leading cause of cancer mortality [1]. Although the median age at diagnosis for cancer is 65 years, about 40 per 100,000 women between 30 and 39 years of age are diagnosed with invasive breast cancer in Germany each year, accounting for 4% of all breast cancer cases [2,3]. A breast cancer diagnosis at a young age may collide with future family planning and raises concerns about the reproductive and obstetric outcome. Additionally, postponing childbearing in modern women to higher ages will lead to an increasing number of breast cancer women with the desire to conceive after cancer treatment. Individual counseling concerning future fertility and possible fertility-sparing treatments is thus paramount for these patients. Indeed, the recently published POSITIVE-Trial reported that the short-term risk for distant recurrence did not worsen after a temporary interruption of endocrine therapy to attempt pregnancy among selected women with hormone receptor-positive breast cancer [4].

Beyond the concerns about cancer prognosis, recent research on pregnancy outcomes revealed increased risks of preterm birth, low birth weight, and cesarean delivery [5,6]. Little is known about further pregnancy complications such as hypertensive disorders, gestational diabetes, or cervical insufficiency, however. International studies in Australia and Sweden indicated an increase in adverse pregnancy outcomes including preeclampsia among adolescent and young adult cancer survivors (AYA) [7,8]. However, the data from grouping various cancer types cannot be extrapolated for breast cancer survivors in general and might conceal risks related to a specific cancer type and treatment [9,10].

In this large observational cohort study, we compared selected adverse pregnancy outcomes among breast cancer survivors to those without a cancer diagnosis based on German statutory health insurance claims data. Furthermore, we evaluated these associations according to the time interval to birth and to treatment type.

## 2. Methods

### 2.1. Data Sources

Based on insurance claims data of the AOK Baden-Württemberg, we identified 94,845 women who were diagnosed with primary invasive breast cancer (ICD-10 code C50) and 97,121 age-matched control patients without a breast cancer diagnosis between 2010 and 2020. Detailed information on the dataset was previously reported [11]. Breast cancer diagnosis was defined by ICD-10 C50 (International Classification of Diseases) during inpatient admission between 2010 and 2020. Births were identified by DRG (diagnosis-related groups) coding and German midwifery coding. The data on cancer treatment were extracted by using ATC (anatomical therapeutic chemical) and OPS (operation and procedure classification system) codes (see Table S1).

Ethical approval was obtained from the Ethics Committee of Tuebingen University Medical Faculty and University Hospital (380/2020BO), and an exemption from requiring informed consent was granted as all data were sufficiently anonymized and cannot be traced by the study team. This study adheres to the STROBE guideline for observational cohort studies and all methods were performed in accordance with the Declaration of Helsinki.

### 2.2. Study Population

A detailed description of the study cohort has already been published [11]. In brief, of all patients with a C50 diagnosis, 2864 women were identified as having delivered at least one infant at any time (see Table S2). We excluded births prior to breast cancer diagnosis and those pregnancies during or directly after cancer diagnosis by setting a minimum time interval between breast cancer diagnosis and delivery at a minimum of three quarters. C50 diagnoses in the outpatient setting were viewed as unreliable and were excluded. A first encoding of C50 between 1 January 2010 and 30 June 2010 or after 1 January 2020 was excluded since the initial diagnosis of breast cancer could not be identified with certainty and a follow-up duration was too short. We excluded those with an overall insurance duration of less than 40%, any other cancer diagnoses except non-melanoma skin cancer, and secondary metastases before C50 coding. Multiple births were counted as one event. Only the first pregnancy within the cohort period was included for analysis. Thus, the final analysis set included 74 breast cancer survivors.

Matching was performed to pair each woman with a history of breast cancer and a postdiagnosis birth with three unique patients in the control group (1:3 ratio). Since age at delivery is an important risk factor for adverse obstetric outcomes, the age at delivery was selected as the main criterion. The final analysis cohort included 74 births to breast cancer survivors compared to 222 births to women without any cancer.

### 2.3. Outcomes

The primary endpoint included adverse pregnancy outcomes during the first postdiagnosis pregnancy, including hypertensive disorders of pregnancy, small-for-gestational age fetuses (SGA), preterm delivery, low birth weight (LBW), gestational diabetes, preterm rupture of membranes, cervical insufficiency, and delivery by cesarean section based on ICD-10 and OPS coding (see Table S1).

### 2.4. Statistical Analysis

A statistical analysis was performed using R version 4.0.2 and R-Studio v. 1.3.1056 for Windows (32/64 bit). Matching was performed using the R package optmatch to find matches that minimize the age differences between case and control patients. The study cohort was primarily stratified into women with a history of breast cancer versus women without any cancer diagnosis. Two-sided *p* values *<* 0.05 were considered significant. Baseline characteristics were stated as absolute and relative counts or as means with standard deviation where appropriate. Odds ratios (OR) with a 95% confidence interval (CI) were calculated using the Baptista–Pike method with *p* values according to χ^2^ test. The group of breast cancer survivors was then stratified according to treatment type and time interval until the first postdiagnosis birth (categorical < 2 years, 2–5 years, >5 years). Kaplan–Meier curves with hazard ratios (HR) and a 95% CI were calculated, and the probability for the first postdiagnosis birth (time until first birth) was compared according to treatment type.

## 3. Results

For the final analysis, 74 patients with a prior breast cancer diagnosis were compared to 222 age-matched women without a previous history of cancer. Table 1 shows the demographic characteristics of the cohort according to the history of breast cancer prior to pregnancy. The mean age at delivery was 35 ± 4.5 years.

The mean age at breast cancer diagnosis was 31.3 ± 4.5 years with a mean time interval until the first birth of 3.8 ± 2.1 years. Most postdiagnosis births were conceived in less than 5 years after cancer diagnosis. The group of mothers with a prior breast cancer diagnosis consisted of 66.2% nulliparous women. Chemotherapy was the most commonly used systemic treatment (66.2%), followed by endocrine (32.4%) and anti-Her2 treatment (18.9%) (Table 1).

**Figure 1:**
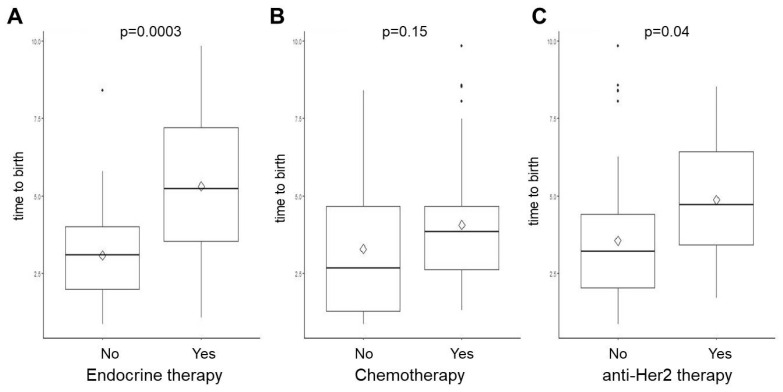
Time to the first birth (years) in BC survivors according to treatment type. Breast cancer patients were stratified by the following treatment types: (**A**) endocrine therapy, (**B**) chemotherapy, and (**C**) anti-Her2 therapy. Box plots represent the median with interquartile range. The mean is shown as rhomb. Whiskers indicate minimum and maximum. Individual dots show outliers.

**Figure 2:**
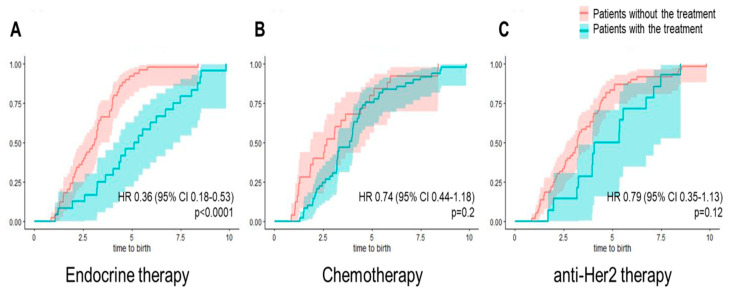
Cumulative incidence of the first birth in BC survivors according to treatment type. Breast cancer survivors with a postdiagnosis birth stratified by endocrine therapy (**A**), chemotherapy (**B**), and anti-Her2 treatment (**C**). Hazard ratios with 95% CI are shown.

The time from breast cancer diagnosis to the first delivery was significantly higher among women who received endocrine treatment (5.3 ± 2.5 years versus 3.1 ± 1.5 years, *p* = 0.0003) and among those who received anti-Her2 targeted therapy (4.9 ± 2.1 years versus 3.6 ± 2.1 years, *p* = 0.04) compared to BC patients without this certain treatment (Figure 1). Endocrine therapy was associated with a significantly lower probability of birth after breast cancer diagnosis compared to breast cancer patients without endocrine therapy (HR 0.36; 95 CI 0.18–0.53; *p* < 0.0001, effect size 0.31) (Figure 2).

Among breast cancer survivors, preterm birth was reported in 9.5% of infants, SGA in 10.8%, hypertensive disorders in 8.1%, and gestational diabetes in 23%. Obstetric outcomes such as hypertensive disorders (OR 0.67; 95% CI 0.26–1.69), preterm birth (OR 0.9; 95% CI 0.37–2.20), gestational diabetes (OR 1.08; 95% CI 0.58–2.03), LBW (OR 2.06; 95% CI 0.85–4.97) and the risk of cesarean section (OR 1.04; 95% CI 0.49–2.18) did not significantly differ between the group of breast cancer survivors compared to women without a prior cancer diagnosis (Table 2).

However, women with a history of breast cancer were at an over threefold increased risk of SGA during the first postdiagnosis pregnancy (OR 3.24; 95% CI 1.17–8.97, *p* = 0.03). This association was even more emphasized when stratifying for the time interval between breast cancer diagnosis and birth: women were at the highest risk for a pregnancy complicated by SGA when the time interval to birth was less than 2 years (OR 5.73; 95% CI 1.37–24.02, *p* = 0.03) compared to women without cancer diagnosis. Similarly, delivery in less than 2 years after breast cancer diagnosis increased the risk for a low birth weight among these infants (OR 4.57; 95% CI 1.32–15.87, *p* = 0.02) (Table 3). The subgroup analysis for the type of breast cancer treatment revealed no significant associations with adverse obstetric outcomes (Table 3).

## 4. Discussion

Here, we present insurance claims data on 74 breast cancer survivors and the obstetric outcome of their first delivery after cancer diagnosis. Based on our analysis, we found no convincing evidence of a generally increased risk of adverse obstetric outcomes, including hypertensive disorders, preterm birth, gestational diabetes, and cesarean section for women with a history of breast cancer. Breast cancer survivors who delivered in less than 2 years after diagnosis were at significantly increased risk for SGA and LBW, however. The time interval until the first postdiagnosis birth was significantly longer in women treated with endocrine therapy.

Continuing advances in therapy have successfully reduced treatment-related adverse long-term effects such as breast-conserving surgery and the limitation of axillary surgery to combat lymphedema. Fertility itself is a crucial part of a woman’s individual quality of life. In addition to cancer prognosis, cancer survivors of reproductive age require appropriate counseling about their future fertility and obstetric risks and options for preserving fertility. The growing number of young breast cancer survivors coincides with current trends towards a first pregnancy at higher ages. In a recent meta-analysis, Arecco et al. highlighted that pregnancy after hormone receptor-positive breast cancer can be considered safe with no impact on disease-free survival and that overall survival was even better [12]. Most cancer survivors will face treatment-induced toxicities, which could possibly modify the future risk of obstetric complications. Preexisting maternal cardiovascular or renal dysfunctions are thought to be involved in the pathogenesis of various adverse obstetric conditions such as preeclampsia and preterm birth [13]. A Swedish register-based study revealed an over threefold increased risk of preeclampsia among 278 AYA cancer survivors in their subsequent pregnancies (aOR 3.46; 95% CI 1.58–7.56) [7]. However, the specific data are scarce on women with a history of breast cancer and their subsequent risk of preeclampsia. In line with our results, retrospective analyses in France and Germany found no substantial association between prior breast cancer diagnosis and later preeclampsia (OR 2.54; 95% CI 0.49–13.32; *p* = 0.268) [14,15]. Surprisingly, our results even revealed a tendency to a lowered risk of hypertensive disorders after BC, which might arise from highly compliant pregnant women with a history of breast cancer in combination with responsible health care practitioners who may be overly aware of potential risks during pregnancy.

The available data in the literature on the risk of preterm delivery in women with a history of breast cancer are conflicting. While results from 165 German breast cancer survivors by Jacob et al. support our data reassuring affected women about their not significantly elevated risk of preterm delivery (OR 2.01; 95% CI 0.18–22.41) [15], a recent population-based study in the United States by Anderson et al. found an elevated prevalence of preterm delivery among 367 women diagnosed with breast cancer (PR 1.98; 95% CI 1.56–2.51) [9]. In contrast to our cohort, Anderson et al. also included cancer diagnoses during pregnancy in their analyses, which constituted 25% of all breast cancer survivors. However, a cancer diagnosis during pregnancy with potential cancer treatment-related toxicity may affect pregnancy outcomes more directly or can itself be a reason for preterm delivery in favor of cancer prognosis and further treatment. These iatrogenic deliveries might be responsible for the immensely high rate of 19% for preterm birth among breast cancer survivors in the Anderson cohort [9]. Among women who conceive after breast cancer diagnosis, the association with preterm delivery appears to be modest or not statistically significant: Based on cancer registries in Georgia, North Carolina, and Tennessee in the USA, Hartnett et al. calculated risk ratios of up to 1.3 (95% CI 1.0–1.8) for preterm deliveries among women with a previous diagnosis of breast cancer [16]. Likewise, another recent study reported a similar prevalence of preterm birth between women with versus without breast cancer (PR 1.10; 95% CI 0.78–1.54) [17]. The reassuring results on preterm birth and hypertension in pregnancy based on our cohort are furthermore supported by the meta-analysis of Lambertini et al., who reported significant, albeit only slightly increased risks for these adverse obstetric outcomes [18].

In our cohort, the time interval from breast cancer diagnosis to the first postdiagnosis birth was significantly higher among women receiving endocrine therapy (5.3 years) and fits with the general recommendation for breast cancer patients to go through endocrine therapy for at least 2 years before planning a pregnancy [19]. Although individual decisions on the timing of family planning cannot be fully excluded, our results are in line with a study conducted by Labrosse et al., who found a significant association between endocrine therapy and the time from diagnosis to pregnancy (42 months versus 61 months) [14]. Furthermore, endocrine therapy seemed to have an independent impact on the time between the pregnancy attempt and the occurrence of pregnancy [14].

The timing of pregnancy seems to be crucial for both the cancer-related prognosis and obstetric risks: in our analyses, women who delivered less than 2 years after breast cancer diagnosis were found to be at a higher risk of SGA and LBW than women without a cancer diagnosis. Mechanisms underlying the development of cancer, such as transient effects on the immune, inflammatory, and vascular system that are thought to be involved in the pathogeneses of prenatal growth retardation, might explain a temporary increase in growth retardation associated with the diagnosis [20].

### Strengths and Limitations

Given the inherent nature of insurance claims data, several limitations need to be addressed: First, the retrospective design implies that the results are explorative rather than confirmatory. Second, diagnoses of breast cancer and obstetric outcomes were based on the available database, which was not primarily created for clinical research purposes and is therefore prone to misclassification bias and missing data. Third, detailed information on health behavior such as smoking, body mass index, and diet as well as cancer stage and histological subtypes was not available. However, we classified the tumor biology based on the prescribed medication: luminal tumors were characterized by prescriptions of endocrine therapy, while Her2 status depended on prescriptions of trastuzumab or pertuzumab or related antibody-drug conjugates. This approach resulted in plausible subtype distributions among young breast cancer patients as reported by others [21]. Lastly, with regard to the small amount of outcome event data, the current results should not be misinterpreted as showing that the outcomes are similar with or without breast cancer. Data from meta-analyses are needed to obtain a clearer answer.

## 5. Conclusions

Our findings indicate that women who consider pregnancy after a history of breast cancer are not generally at increased risk of adverse obstetric outcomes, including hypertensive disorders, preterm birth, gestational diabetes, and cesarean section. However, the timing of pregnancy seems to be critical; delivery in less than 2 years after diagnosis may be associated with an increased risk for SGA and LBW. These data could help fertility counseling before and after cancer treatments.

## Figures and Tables

**Table 1:** Background characteristics of women with and without a history of breast cancer and breast cancer details.

	Mothers Exposed to Breast Cancer		Mothers Not Exposed to Cancer	
Total	74	100%	222	100%
Age at delivery	35.1	±4.5	35.1	±4.4
Mother’s highest educational degree				
Bachelor	6	10.5%	6	3.9%
Master	4	7%	17	11.2%
Doctorate	1	1.8%	3	2%
Other	12	16.2%	21	9%
Use of assisted reproductive technology	9	12.2%	35	15.8%
Multiple gestation	5	6.8%	15	6.8%
Mode of delivery				
Spontaneous	29	39.2%	58	26.1%
Assisted vaginal delivery	2	2.7%	9	4.1%
Cesarean section	11	14.9%	32	14.4%
Breast cancer subtype				
HR negative, Her2 negative	42	56.7%		
HR negative, Her2 positive	8	10.8%		
HR positive, Her2 negative	18	24%		
HR positive, Her2 positive	6	8%		
Treatment				
Chemotherapy	49	66.2%		
Endocrine therapy	24	32.4%		
Anti-Her2 therapy	14	18.9%		
Breast surgery	70	94.6%		
Mean age at breast cancer diagnosis (years)	31.3	±4.5		
Time to first birth after diagnosis (years)	3.8	±2.1		
Time between diagnosis and first postdiagnosis birth				
<2 years	17	23%		
2-5 years	40	54.1%		
>5 years	17	23%		

Data are presented as mean ± standard deviation or as absolute and relative numbers. HR: hormone receptor.

**Table 2:** Selected obstetric outcomes for the first birth conceived after breast cancer diagnosis compared to women without a previous cancer diagnosis.

	No Cancer		Breast Cancer Survivor			
	n	%	n	%	OR (95% CI)	*p* Value
Hypertensive disorders	26	11.7	6	8.1	0.67 (0.26; 1.69)	0.52
Small for gestational age	8	3.6	8	10.8	3.24 (1.17; 8.97)	0.03
Preterm birth	23	10.4	7	9.5	0.90 (0.37; 2.20)	1
Low birth weight	14	6.3	9	12.2	2.06 (0.85; 4.97)	0.17
Gestational diabetes	48	21.6	17	23	1.08 (0.58; 2.03)	0.94
Premature rupture of the membranes	57	25.7	23	31.1	1.31 (0.73; 2.32)	0.45
Cervical insufficiency	17	7.7	7	9.5	1.26 (0.50; 3.17)	0.81
Large for gestational age	10	4.5	5	6.8	1.54 (0.51; 4.65)	0.55
Cesarean section	32	14.4	11	14.9	1.04 (0.49; 2.18)	1

Data are expressed as odds ratios (OR) with 95% confidence interval (CI). *p* values calculated using χ^2^ test.

**Table 3:** Subgroup analyses of selected obstetric outcomes for the first birth conceived after breast cancer diagnosis compared to women without a previous cancer diagnosis.

	Endocrine Therapy OR (95% CI)	Chemotherapy	Time Interval Until First Postdiagnosis Birth		
		OR (95% CI)	<2 Years OR (95% CI)	2–5 Years OR (95% CI)	>5 Years OR (95% CI)
Hypertensive disorders	0.69 (0.15; 3.08)	0.86 (0.31; 2.36)	NA	1.08 (0.39; 2.99)	0.47 (0.06; 3.70)
Small for gestational age	1.16 (0.14; 9.72)	1.74 (0.53; 6.83)	5.73 (1.37; 24.02)	3.82 (1.18; 12.35)	NA
Preterm birth	0.79 (0.17; 3.56)	0.56 (0.16; 1.96)	1.85 (0.5; 6.94)	0.70 (0.20; 2.46)	0.54 (0.07; 4.24)
Low birth weight	0.65 (0.08; 5.14)	0.97 (0.27; 3.51)	4.57 (1.32; 15.87)	2.12 (0.72; 6.26)	NA
Gestational diabetes	1.21 (0.45; 3.21)	1.18 (0.57; 2.43)	0.78 (0.21; 2.81)	1.05 (0.47; 2.36)	1.51 (0.51; 4.5)
Premature rupture of the membranes	0.76 (0.27; 2.13)	1.28 (0.65; 2.52)	1.58 (0.56; 4.46)	0.96 (0.44; 2.1)	2.03 (0.74; 5.57)
Cervical insufficiency	NA	1.07 (0.34; 3.34)	1.61 (0.34; 7.62)	1.34 (0.43; 4.21)	0.75 (0.09; 6.03)
Large for gestational age	NA	1.38 (0.37; 5.22)	NA	1.72 (0.45; 6.54)	2.83 (0.57; 14.08)
Cesarean section	0.85 (0.24; 3.01)	0.99 (0.41; 2.39)	1.83 (0.56; 5.96)	0.66 (0.22; 1.98)	1.27 (0.35; 4.68)

Data are expressed as odds ratios (OR) with 95% confidence interval. *p* values calculated using χ^2^ test. NA: not applicable due to small case number.

## Data Availability

The data that support the findings of this study were provided by AOK Baden-Wuerttemberg, but restrictions apply to the availability of these data, which were used under license for the current study and thus are not publicly available. The data are, however, available from the authors upon reasonable request and with permission of AOK Baden-Wuerttemberg.

## Supplementary Materials

The following supporting information can be downloaded at: https://www.mdpi.com/article/10.3390/cancers16183230/s1. Table S1: definition of variables; Table S2: inclusion and exclusion criteria of the case and control group.
