# Supplementary material for: Adverse Obstetric Outcomes after Breast Cancer Diagnosis: An Observational Database Study in Germany
Source: Cancers (Basel). 2024 Sep 22;16(18):3230. doi: 10.3390/cancers16183230 (PMC11430513; doi:10.3390/cancers16183230)
Supplement: Supplementary file 1 [file cancers-16-03230-s001.zip › cancers-3169316-supplementary.pdf]

Table S1: Definitions of variables

	ICD-10 codes	OPS	ATC Codes
Breast cancer	C50		
Endocrine therapy			L02BA, L02BG
Chemotherapy		8-54	L01AA01, L01BC, L01CD, L01XA, L01DB
Anti-Her2 treatment		6-001, 6-007, 6-00f	L01FD, L01XC03, L01XC13, L01XC14, L01XE07, L01XH02
Breast surgery		5-87, 5-88	
Hypertensive disorders	O11, O13, O14, O15		
Small for gestational age	P05		
Preterm birth	O60.1, P07		
Low birth weight	P05, P07		
Gestational diabetes	O24.4		
Premature rupture of the membranes	O42		
Cervical insufficiency	O34.3		
Large for gestational age	P08.0, P08.1, O36.6		
Multiple gestation			
Vaginal delivery	O80	5-725	
Assisted vaginal delivery	O81	5-720, 5-724, 5-728, 5-729, 5-738, 5-734	
Cesarean section	O82	5-740, 5-741, 5-742	
Preexisting Hypertension	I10.-, I12.-, I15.-		
Cardiovascular disease	I20.-, I21, I22.-, I23.-, I24.-, I25.-, I51.6, I51.8; I51.9, I13.-, I11.-		
Chronic kidney disease	N18.-, N19, Z49.1, Z49.2, Z94.0, Z99.2		
Cerebrovascular disease	G45.-, I60.-, I61.-, I62.-, I63.-, I64.-, I65.-, I66.-, I67.-, I69.-		
Diabetes mellitus	E10.-, E11.-, E12.-, E13.- O24.0, O24.1, O24.2, O24.3		
Smoking	Z72.0		
Assisted reproductive technology	Z31.1, Z31.2, ZZ31.3, Z31.8, Z31.9, N98		
Obesity, dyslipidemia	E66.- E78.-		

ICD-10: International Classification of Diseases; ATC: Anatomical Therapeutic Chemical;

OPS: Operation and procedure classification system.

Table S2: Inclusion and exclusion criteria of the case and control group

	Breast cancer		Control	
	**n**	**(%)**	**n**	**(%)**
Total deliveries	2864	100	3122	100
First delivery >0.75 years after diagnosis	1495	52.2	1240	39.7
Excluded unreliable C50 diagnoses	1181	41.2	0	0
Excluded diagnoses outside time window	146	5.1	0	0
Excluded secondary neoplasia	74	2.6	40	1.3
Excluded metastases	9	0.3	9	0.3
Excluded insured too short	11	0.4	97	3.1
Excluded due to matching	0	0	773	0
Included in final analysis set	74	2.6	222	7.1
